# Supplementary material for: HIVprotI: an integrated web based platform for prediction and design of HIV proteins inhibitors
Source: J Cheminform. 2018 Mar 9;10:12. doi: 10.1186/s13321-018-0266-y (PMC5845081; doi:10.1186/s13321-018-0266-y)
Supplement: Supplementary file 2 — Additional file 2. Source code of HIVProtI web server. [file 13321_2018_266_MOESM2_ESM.zip › HIVprotI_Source-code/hivprotI/weka-3-6-6/doc/weka/experiment/RemoteExperiment.html]

RemoteExperiment


---


|  |  |  |
| --- | --- | --- |
| **Overview** \| **Package** \| **Class** \| **Tree** \| **Deprecated** \| **Index** \| **Help** \| **Weka's home** | |  |
| **PREV CLASS**   **NEXT CLASS** | **FRAMES**    **NO FRAMES**     **All Classes** |  |
| SUMMARY: NESTED \| FIELD \| CONSTR \| METHOD | DETAIL: FIELD \| CONSTR \| METHOD |  |


---


## weka.experiment Class RemoteExperiment

```
java.lang.Object
  weka.experiment.Experiment
      weka.experiment.RemoteExperiment
```

**All Implemented Interfaces:**: java.io.Serializable, OptionHandler, RevisionHandler

---

``` public class RemoteExperiment extends Experiment ```

Holds all the necessary configuration information for a distributed
experiment. This object is able to be serialized for storage on disk.

This class is experimental at present. Has been tested using
CSVResultListener (sending results to standard out) and
DatabaseResultListener (InstantDB + RmiJdbc bridge).

Getting started:

Start InstantDB (with the RMI bridge) on some machine. If using java2
then specify -Djava.security.policy=db.policy to the
virtual machine. Where db.policy is as follows:   

```
 grant {
   permission java.security.AllPermission;
 };
```

Start RemoteEngine servers on x machines as per the instructons in the
README\_Experiment\_Gui file. There must be a
DatabaseUtils.props in either the HOME or current directory of each
machine, listing all necessary jdbc drivers.

The machine where a RemoteExperiment is started must also have a copy
of DatabaseUtils.props listing the URL to the machine where the
database server is running (RmiJdbc + InstantDB).

Here is an example of starting a RemoteExperiment:

```
 java -Djava.rmi.server.codebase=file:/path to weka classes/ \
 weka.experiment.RemoteExperiment -L 1 -U 10 \
 -T /home/ml/datasets/UCI/iris.arff \
 -D "weka.experiment.DatabaseResultListener" \
 -P "weka.experiment.RandomSplitResultProducer" \
 -h rosebud.cs.waikato.ac.nz -h blackbird.cs.waikato.ac.nz -r -- \
 -W weka.experiment.ClassifierSplitEvaluator -- \
 -W weka.classifiers.bayes.NaiveBayes
```

The "codebase" property tells rmi where to serve up weka classes from.
This can either be a file url (as long as a shared file system is being
used that is accessable by the remoteEngine servers), or http url (which
of course supposes that a web server is running and you have put your
weka classes somewhere that is web accessable). If using a file url the
trailing "/" is \*most\* important unless the weka classes are in a jar
file.

Valid options are:

```
 -L <num>
  The lower run number to start the experiment from.
  (default 1)
```

```
 -U <num>
  The upper run number to end the experiment at (inclusive).
  (default 10)
```

```
 -T <arff file>
  The dataset to run the experiment on.
  (required, may be specified multiple times)
```

```
 -P <class name>
  The full class name of a ResultProducer (required).
  eg: weka.experiment.RandomSplitResultProducer
```

```
 -D <class name>
  The full class name of a ResultListener (required).
  eg: weka.experiment.CSVResultListener
```

```
 -N <string>
  A string containing any notes about the experiment.
  (default none)
```

```
 Options specific to result producer weka.experiment.RandomSplitResultProducer:
```

```
 -P <percent>
  The percentage of instances to use for training.
  (default 66)
```

```
 -D
 Save raw split evaluator output.
```

```
 -O <file/directory name/path>
  The filename where raw output will be stored.
  If a directory name is specified then then individual
  outputs will be gzipped, otherwise all output will be
  zipped to the named file. Use in conjuction with -D. (default splitEvalutorOut.zip)
```

```
 -W <class name>
  The full class name of a SplitEvaluator.
  eg: weka.experiment.ClassifierSplitEvaluator
```

```
 -R
  Set when data is not to be randomized and the data sets' size.
  Is not to be determined via probabilistic rounding.
```

```
 Options specific to split evaluator weka.experiment.ClassifierSplitEvaluator:
```

```
 -W <class name>
  The full class name of the classifier.
  eg: weka.classifiers.bayes.NaiveBayes
```

```
 -C <index>
  The index of the class for which IR statistics
  are to be output. (default 1)
```

```
 -I <index>
  The index of an attribute to output in the
  results. This attribute should identify an
  instance in order to know which instances are
  in the test set of a cross validation. if 0
  no output (default 0).
```

```
 -P
  Add target and prediction columns to the result
  for each fold.
```

```
 Options specific to classifier weka.classifiers.rules.ZeroR:
```

```
 -D
  If set, classifier is run in debug mode and
  may output additional info to the console
```

**Version:**
:   $Revision: 1.16 $

**Author:**
:   Mark Hall (mhall@cs.waikato.ac.nz)

**See Also:**: Serialized Form

---

**Field Summary**

**Fields inherited from class weka.experiment.Experiment**

`FILE_EXTENSION`


**Constructor Summary**

`RemoteExperiment()`
  
          Construct a new RemoteExperiment using an empty Experiment as base
Experiment

`RemoteExperiment(Experiment base)`
  
          Construct a new RemoteExperiment using a base Experiment


| **Method Summary** | |
| --- | --- |
| `void` | `abortExperiment()`             Set the abort flag |
| `void` | `addRemoteExperimentListener(RemoteExperimentListener r)`             Add an object to the list of those interested in recieving update information from the RemoteExperiment |
| `void` | `addRemoteHost(java.lang.String hostname)`             Add a host name to the list of remote hosts |
| `void` | `advanceCounters()`             overides the one in Experiment |
| `Experiment` | `getBaseExperiment()`             Get the base experiment used by this remote experiment |
| `javax.swing.DefaultListModel` | `getRemoteHosts()`             Get the list of remote host names |
| `java.lang.String` | `getRevision()`             Returns the revision string. |
| `boolean` | `getSplitByDataSet()`             Returns true if sub experiments are to be created on the basis of data set.. |
| `void` | `initialize()`             Prepares a remote experiment for running, creates sub experiments |
| `void` | `launchNext(int wexp, int ah)`             Launch a sub experiment on a remote host |
| `static void` | `main(java.lang.String[] args)`             Configures/Runs the Experiment from the command line. |
| `void` | `nextIteration()`             Overides the one in Experiment |
| `void` | `postProcess()`             overides the one in Experiment |
| `void` | `runExperiment()`             Overides runExperiment in Experiment |
| `void` | `setBaseExperiment(Experiment base)`             Set the base experiment. |
| `void` | `setDatasets(javax.swing.DefaultListModel ds)`             Set the datasets to use in the experiment |
| `void` | `setNotes(java.lang.String newNotes)`             Set the user notes. |
| `void` | `setPropertyArray(java.lang.Object newPropArray)`             Sets the array of values to set the custom property to. |
| `void` | `setPropertyPath(PropertyNode[] newPropertyPath)`             Sets the path of properties taken to get to the custom property to iterate over. |
| `void` | `setRemoteHosts(javax.swing.DefaultListModel list)`             Set the list of remote host names |
| `void` | `setResultListener(ResultListener newResultListener)`             Sets the result listener where results will be sent. |
| `void` | `setResultProducer(ResultProducer newResultProducer)`             Set the result producer used for the current experiment. |
| `void` | `setRunLower(int newRunLower)`             Set the lower run number for the experiment. |
| `void` | `setRunUpper(int newRunUpper)`             Set the upper run number for the experiment. |
| `void` | `setSplitByDataSet(boolean sd)`             Set whether sub experiments are to be created on the basis of data set. |
| `void` | `setUsePropertyIterator(boolean newUsePropertyIterator)`             Sets whether the custom property iterator should be used. |
| `java.lang.String` | `toString()`             Overides toString in Experiment |

**Methods inherited from class weka.experiment.Experiment**

`classFirst, getAdvanceDataSetFirst, getCurrentDatasetNumber, getCurrentPropertyNumber, getCurrentRunNumber, getDatasets, getNotes, getOptions, getPropertyArray, getPropertyArrayLength, getPropertyArrayValue, getPropertyPath, getResultListener, getResultProducer, getRunLower, getRunUpper, getUsePropertyIterator, hasMoreIterations, listOptions, read, setAdvanceDataSetFirst, setOptions, write`

**Methods inherited from class java.lang.Object**

`equals, getClass, hashCode, notify, notifyAll, wait, wait, wait`

**Constructor Detail**

### RemoteExperiment

```
public RemoteExperiment()
                 throws java.lang.Exception
```

:   Construct a new RemoteExperiment using an empty Experiment as base
    Experiment

    **Throws:**: `java.lang.Exception` - if the base experiment is null

---


### RemoteExperiment

```
public RemoteExperiment(Experiment base)
                 throws java.lang.Exception
```

:   Construct a new RemoteExperiment using a base Experiment

    **Parameters:**: `base` - the base experiment to use **Throws:**: `java.lang.Exception` - if the base experiment is null


**Method Detail**

### getSplitByDataSet

```
public boolean getSplitByDataSet()
```

:   Returns true if sub experiments are to be created on the basis of
    data set..

    :   **Returns:**: a `boolean` value indicating whether sub experiments are to be created on the basis of data set (true) or run number (false).

---


### setSplitByDataSet

```
public void setSplitByDataSet(boolean sd)
```

:   Set whether sub experiments are to be created on the basis of
    data set.

    :   **Parameters:**: `sd` - true if sub experiments are to be created on the basis of data set. Otherwise sub experiments are created on the basis of run number.

---


### addRemoteExperimentListener

```
public void addRemoteExperimentListener(RemoteExperimentListener r)
```

:   Add an object to the list of those interested in recieving update
    information from the RemoteExperiment

    :   **Parameters:**: `r` - a listener

---


### getBaseExperiment

```
public Experiment getBaseExperiment()
```

:   Get the base experiment used by this remote experiment

    :   **Returns:**: the base experiment

---


### setBaseExperiment

```
public void setBaseExperiment(Experiment base)
                       throws java.lang.Exception
```

:   Set the base experiment. A sub experiment will be created for each
    run in the base experiment.

    :   **Parameters:**: `base` - the base experiment to use. **Throws:**: `java.lang.Exception` - if supplied base experiment is null

---


### setNotes

```
public void setNotes(java.lang.String newNotes)
```

:   Set the user notes.

    :   **Overrides:**: `setNotes` in class `Experiment`
    :   **Parameters:**: `newNotes` - New user notes.

---


### setRunLower

```
public void setRunLower(int newRunLower)
```

:   Set the lower run number for the experiment.

    :   **Overrides:**: `setRunLower` in class `Experiment`
    :   **Parameters:**: `newRunLower` - the lower run number for the experiment.

---


### setRunUpper

```
public void setRunUpper(int newRunUpper)
```

:   Set the upper run number for the experiment.

    :   **Overrides:**: `setRunUpper` in class `Experiment`
    :   **Parameters:**: `newRunUpper` - the upper run number for the experiment.

---


### setResultListener

```
public void setResultListener(ResultListener newResultListener)
```

:   Sets the result listener where results will be sent.

    :   **Overrides:**: `setResultListener` in class `Experiment`
    :   **Parameters:**: `newResultListener` - the result listener where results will be sent.

---


### setResultProducer

```
public void setResultProducer(ResultProducer newResultProducer)
```

:   Set the result producer used for the current experiment.

    :   **Overrides:**: `setResultProducer` in class `Experiment`
    :   **Parameters:**: `newResultProducer` - result producer to use for the current experiment.

---


### setDatasets

```
public void setDatasets(javax.swing.DefaultListModel ds)
```

:   Set the datasets to use in the experiment

    :   **Overrides:**: `setDatasets` in class `Experiment`
    :   **Parameters:**: `ds` - the list of datasets to use

---


### setUsePropertyIterator

```
public void setUsePropertyIterator(boolean newUsePropertyIterator)
```

:   Sets whether the custom property iterator should be used.

    :   **Overrides:**: `setUsePropertyIterator` in class `Experiment`
    :   **Parameters:**: `newUsePropertyIterator` - true if so

---


### setPropertyPath

```
public void setPropertyPath(PropertyNode[] newPropertyPath)
```

:   Sets the path of properties taken to get to the custom property
    to iterate over.

    :   **Overrides:**: `setPropertyPath` in class `Experiment`
    :   **Parameters:**: `newPropertyPath` - an array of PropertyNodes

---


### setPropertyArray

```
public void setPropertyArray(java.lang.Object newPropArray)
```

:   Sets the array of values to set the custom property to.

    :   **Overrides:**: `setPropertyArray` in class `Experiment`
    :   **Parameters:**: `newPropArray` - a value of type Object which should be an array of the appropriate values.

---


### initialize

```
public void initialize()
                throws java.lang.Exception
```

:   Prepares a remote experiment for running, creates sub experiments

    :   **Overrides:**: `initialize` in class `Experiment`
    :   **Throws:**: `java.lang.Exception` - if an error occurs

---


### abortExperiment

```
public void abortExperiment()
```

:   Set the abort flag

---


### launchNext

```
public void launchNext(int wexp,
                       int ah)
```

:   Launch a sub experiment on a remote host

    :   **Parameters:**: `wexp` - the index of the sub experiment to launch: `ah` - the index of the available host to launch on

---


### nextIteration

```
public void nextIteration()
                   throws java.lang.Exception
```

:   Overides the one in Experiment

    :   **Overrides:**: `nextIteration` in class `Experiment`
    :   **Throws:**: `java.lang.Exception` - never throws an exception

---


### advanceCounters

```
public void advanceCounters()
```

:   overides the one in Experiment

    :   **Overrides:**: `advanceCounters` in class `Experiment`

---


### postProcess

```
public void postProcess()
```

:   overides the one in Experiment

    :   **Overrides:**: `postProcess` in class `Experiment`

---


### addRemoteHost

```
public void addRemoteHost(java.lang.String hostname)
```

:   Add a host name to the list of remote hosts

    :   **Parameters:**: `hostname` - the host name to add to the list

---


### getRemoteHosts

```
public javax.swing.DefaultListModel getRemoteHosts()
```

:   Get the list of remote host names

    :   **Returns:**: the list of remote host names

---


### setRemoteHosts

```
public void setRemoteHosts(javax.swing.DefaultListModel list)
```

:   Set the list of remote host names

    :   **Parameters:**: `list` - the list of remote host names

---


### toString

```
public java.lang.String toString()
```

:   Overides toString in Experiment

    :   **Overrides:**: `toString` in class `Experiment`
    :   **Returns:**: a description of this remote experiment

---


### runExperiment

```
public void runExperiment()
```

:   Overides runExperiment in Experiment

    :   **Overrides:**: `runExperiment` in class `Experiment`

---


### getRevision

```
public java.lang.String getRevision()
```

:   Returns the revision string.

    :   **Specified by:**: `getRevision` in interface `RevisionHandler` **Overrides:**: `getRevision` in class `Experiment`
    :   **Returns:**: the revision

---


### main

```
public static void main(java.lang.String[] args)
```

:   Configures/Runs the Experiment from the command line.

    :   **Parameters:**: `args` - command line arguments to the Experiment.


---


|  |  |  |
| --- | --- | --- |
| **Overview** \| **Package** \| **Class** \| **Tree** \| **Deprecated** \| **Index** \| **Help** \| **Weka's home** | |  |
| **PREV CLASS**   **NEXT CLASS** | **FRAMES**    **NO FRAMES**     **All Classes** |  |
| SUMMARY: NESTED \| FIELD \| CONSTR \| METHOD | DETAIL: FIELD \| CONSTR \| METHOD |  |


---
